# Surface-Engineered Inorganic Nanoporous Membranes for Vapor and Pervaporative Separations of Water–Ethanol Mixtures †

**DOI:** 10.3390/membranes8040095

**Published:** 2018-10-12

**Authors:** Michael Z. Hu, Chaiwat Engtrakul, Brian L. Bischoff, Mi Lu, Mussie Alemseghed

**Affiliations:** 1 Oak Ridge National Laboratory, Oak Ridge, TN 37831, USA; bischoffbl@ornl.gov (B.L.B.); lum1@ornl.gov (M.L.); alemseghed@yahoo.com (M.A.); 2 National Renewable Energy Laboratory, Golden, CO 80401, USA; Chaiwat.Engtrakul@nrel.gov

**Keywords:** inorganic membranes, nanoporous membranes, superhydrophobic, superhydrophilic, ethanol separations, dewatering, dehydration

## Abstract

Surface wettability-tailored porous ceramic/metallic membranes (in the tubular and planar disc form) were prepared and studied for both vapor-phase separation and liquid pervaporative separations of water-ethanol mixtures. Superhydrophobic nanoceramic membranes demonstrated more selective permeation of ethanol (relative to water) by cross-flow pervaporation of liquid ethanol–water mixture (10 wt % ethanol feed at 80 °C). In addition, both superhydrophilic and superhydrophobic membranes were tested for the vapor-phase separations of water–ethanol mixtures. Porous inorganic membranes having relatively large nanopores (up to 8-nm) demonstrated good separation selectivity with higher permeation flux through a non-molecular-sieving mechanism. Due to surface-enhanced separation selectivity, larger nanopore-sized membranes (~5–100 nm) can be employed for both pervaporation and vapor phase separations to obtain higher selectivity (e.g., permselectivity for ethanol of 13.9 during pervaporation and a vapor phase separation factor of 1.6), with higher flux due to larger nanopores than the traditional size-exclusion membranes (e.g., inorganic zeolite-based membranes having sub-nanometer pores). The prepared superhydrophobic porous inorganic membranes in this work showed good thermal stability (i.e., the large contact angle remains the same after 300 °C for 4 h) and chemical stability to ethanol, while the silica-textured superhydrophilic surfaced membranes can tolerate even higher temperatures. These surface-engineered metallic/ceramic nanoporous membranes should have better high-temperature tolerance for hot vapor processing than those reported for polymeric membranes.

## 1. Introduction

While there are many alternative energy sources starting to emerge for stationary power, the transportation energy market is primarily driven by the volatile price of fossil fuels. The conversion of biomass to biofuels represents a vital option for producing a liquid renewable green energy source. The United States’ energy consumption is projected to rise by about 12% from 2012 to 2040. Renewable liquid fuels are projected to have the largest increase in production for meeting the United States’ energy consumption demands—growing from 8% in 2010 to more than 14% of liquid fuels in 2035 [1]. Biochemical conversion [2,3], such as fermentation, is a major pathway for transforming solid biomass or sugar to liquid fuel, such as ethanol.

Challenges exist to integrate separation technologies into the fermentation process to directly produce high-quality ethanol with high efficiency. For example, removing inhibitors (such as n-butanol) from the fermentation broth can increase ethanol production [4]. Additionally, ethanol produced at low concentrations in the bio-fermentation broth needs to be efficiently concentrated and then dehydrated (purified) to qualify as a gasoline blend. The traditional distillation process utilized to concentrate ethanol is energy-intensive and has an azeotropic limitation for ethanol purification [5]. The separation of ethanol from water represents a significant portion of the total operating cost for producing bio-ethanol [6,7]. There has been a good amount of research effort in developing membranes for pervaporative separation of ethanol from ethanol–water liquid mixtures [8,9,10,11,12,13,14,15,16,17,18,19,20,21,22,23,24]. One major class of *water-selective* membranes are focused on the dehydration of water-alcohol mixtures by the pervaporation of liquid mixtures. The types of pervaporation dehydration membranes include zeolites and meta-organic frameworks [8,9,10,11,12,13], ceramics and microporous silica [14,15,16], ceramic-organic hybrids [17,18,19], polymers [20], polymer-inorganic composites or mixed matrices [21,22,23], and microporous carbons [24]. However, very limited dehydration studies on vapor-phase water-alcohol mixtures have been completed in recent years [25]. Several inorganic membrane options for liquid ethanol–water separations exist and are typically zeolite-based and contain sub-nanometer pores to achieve high ethanol selectivity [26]. A small pore size (typically <1 nm) is generally employed to ensure high selectivity in the porous membrane through a size exclusion mechanism. Small pore sizes will tend to limit the vapor or liquid permeation flux, since flux is proportional to the square of the pore diameter. The low permeation flux (for either water or ethanol) makes these size exclusion membranes impractical for many commercial applications. Membranes suitable for moist hot-vapor separations (>250 °C, above which most polymers/zeolites are thermally decomposed) are rarely developed. Here we report new vapor separation data with superhydrophilic surface-tailored nanoporous inorganic tubular membranes with larger pores and good thermal and chemical stabilities.

In the case of separating ethanol–water solutions (such as a fermentation broth), *ethanol-selective* membranes that can extract ethanol out of the dilute solutions would be preferred and more efficient. Most of the hydrophobic membranes in the literature for ethanol pervaporation have so far considered materials such as zeolites/silicalite and their supported thin layers [27,28,29,30,31], polymers such as polydimethylsiloxane (PDMS) [32], polymer-zeolite hybrids [33,34,35], and polymer-inorganic mixed matrices [36,37,38,39]. Recently, some works have considered superhydrophobicity for membrane separations of ethanol–water mixtures [40,41].

**Figure 1:**
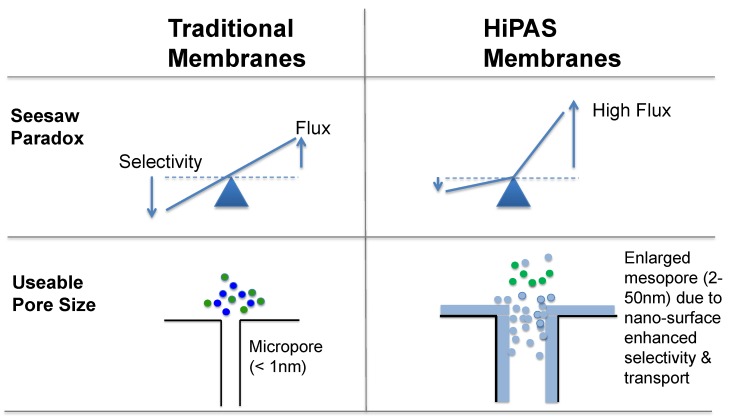
Schematic illustrating the pore size and surface difference between traditional membranes and HiPAS membranes. The HiPAS membranes are designed to take full advantage of the membrane surface superhydrophilicity or superhydrophobicity with relatively larger pores (>a few nanometers to tens of nanometers), to enhance permeation flux with the least loss of selectivity.

In this paper, we report a new class of surface-engineered inorganic (ceramic or metallic) nanoporous membranes of relatively large nanopores (5–100 nm range), i.e., high-performance architecture surface selective (HiPAS) membranes in tubular or planar form. The HiPAS membranes are designed to take full advantage of the membrane surface superhydrophilicity or superhydrophobicity on larger nanopore metallic/ceramic materials (pore size as compared to zeolite or microporous silica materials). Figure 1 illustrates the major conceptual difference of our HiPAS membranes from traditional membranes. Such high-flux membranes have been described in details in our previously published papers, which have studied pyrolysis bio-oil processing related separation applications [42,43]. Here, we focus on the initial exploration of HiPAS nanoporous membranes for ethanol–water separations: i.e., vapor-phase dehydration by superhydrophilic membranes and liquid pervaporation recovery of ethanol by superhydrophobic membranes.

## 2. Experimental

### 2.1. Preparation of Membranes

Porous membrane substrates (as supports) include planar ceramic discs (alumina, ~90 nm averaged pore size) and metallic tubes (stainless steels). The round flat ceramic disc substrates (22-mm diameter), made from alumina powder, were formed using a hydraulic press and had a pore size of 55–60 nm. The pore size of the alumina discs can be tailored by adjusting the sintering temperature. The porous tubular substrates (typically 9-in long) were Type 434 stainless steel (SS) with an average pore size of ~4.3 µm. In some cases, a mesoporous alumina layer was further coated on the inner wall of the SS434 tube and the pore size was adjusted by controlling the sintering temperature of the alumina layer (typically 4-nm, 6-nm, or 8-nm).

The surface of the ceramic discs or inner wall of the above tubes were then chemically functionalized by hydrophobic or hydrophilic ligand molecules. In some cases, before the chemical functionalization, the top surfaces were textured by depositing diatomaceous earth on ceramic discs [44] or by depositing silica aerogel nanoparticles on the inner wall of alumina-modified SS tubes. The surface texture generally boosts the surface effect by effectively adding a range of contact angles to the membrane surface. This helps to make a hydrophobic surface superhydrophobic (with a contact angle >150°) or can increase the degree of hydrophilicity (i.e., superhydrophilic with faster water penetration feature).

The surface-textured discs or tubes were then functionalized with either a hydrophobic silane ligand precursor using a liquid solution treatment process [45] or a hydrophilic chemical solution (such as Hydrophil-S or hydroxyl-terminated silane chemical). Additional details on the fabrication of the HiPAS membranes can be found in our recent publications [42,43]. Specifically, the membranes prepared and tested in this paper are summarized in Table 1.

### 2.2. Characterization of Membrane Materials

At each stage of the membrane fabrication and coating process, the membrane materials were characterized: the support substrate, the intermediate porous modification coating layer, and top textured functional surface. The support substrates (ceramic discs, bare or alumina coated SS434 tubes) are porous and air flux measurements were made over of range of pressures to calculate the air permeance and average pore size of the substrates. A standard isopropanol leak test was used to confirm the absence of leaks or defects [46]. After surface texturing or chemical functionalization, the membrane surface wetting properties were checked by measuring the contact angle or penetration rate after applying a ~3 mm diameter water droplet.

### 2.3. Membrane Separation Study on Dehydration of Ethanol–Water Mixed Vapors

A detailed description of the vapor-phase separation evaluation of the tubular membranes was described elsewhere [43]. Briefly, a horizontal quartz reactor was connected to a custom-built stainless-steel membrane holder housing a tubular membrane. The permeate line of the membrane holder was connected to a molecular beam mass spectrometer (MBMS) to monitor changes in the permeate vapors. The feed mixture consisting of ethanol and water was introduced into a horizontal quartz reactor via syringe pumps (NE-1000, New Era Pump Systems Inc., Farmingdale, NY, USA) and vaporized using a five-zone furnace. The ethanol–water (50:50 mixture by volume) mixture was fed at a constant rate (60 µL/min) into the reactor housed in a furnace at 200 °C. The ethanol–water vapors were transported through the inner tube of the quartz reactor by 0.4 slm helium. This stream was further diluted with a 1.6 slm helium stream from the outer tube of the quartz reactor to allow for proper mixing of the vapors. The membrane holder and permeate line were heated to 200 °C up to the faceplate of MBMS. A small amount of argon was used as a tracer gas (30 sccm) and introduced in the helium carrier gas stream.

### 2.4. Membrane Separation Study on Liquid Ethanol–Water Mixtures by Ethanol Pervaporation

**Figure 2:**
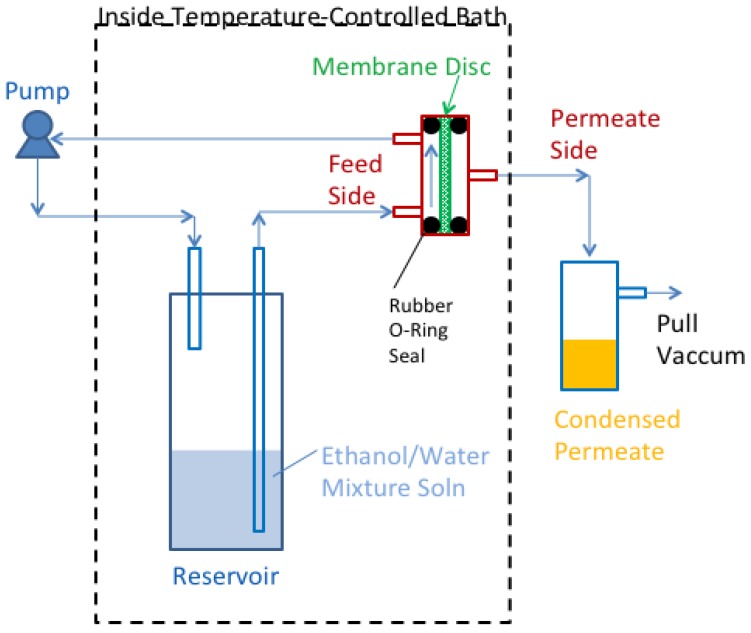
Liquid-phase separation of an ethanol–water solution with a superhydrophobic HiPAS membrane (“Baseline Disc” and “SO Disc”). Schematic for the cross-flow pervaporation separation test setup.

For the liquid ethanol–water solution separation test, a planar disc type membrane setup was used (Figure 2). The solution in the system was maintained at ~80 °C. The solution was pumped out of a reservoir tank into the “feed side” of the membrane disc assembly, and then recirculated back to the reservoir. Samples were taken out of the reservoir over the course of the test and analyzed for ethanol concentration using a standard refractive index method. A mild vacuum (3.9 psia) was applied to the “permeate side” of the membrane disc-holder assembly to help the pervaporation of ethanol across the membrane. The permeate stream was condensed and collected by a cold trap and analyzed for ethanol concentration.

## 3. Results and Discussion

**Figure 3:**
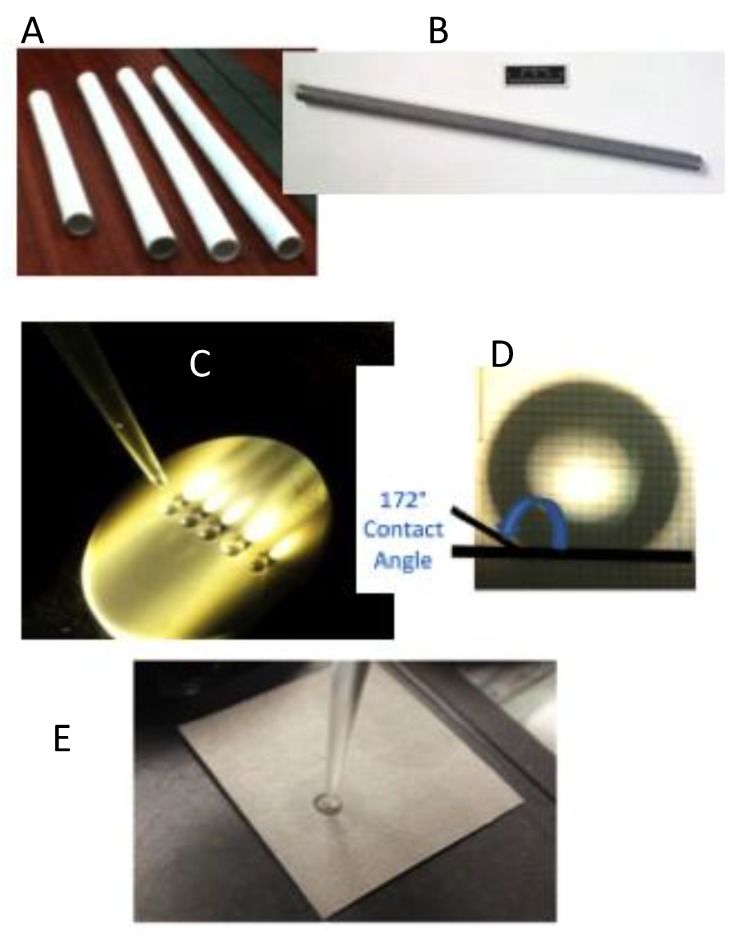
Photos of HiPAS membranes on various inorganic porous support platforms. (**A**) Ceramic tubes, (**B**) a metallic SS434 tube, with an inner wall modified with superhydrophilic deposit textures, (**C**) a ceramic (alumina) disc coated with superhydrophobic surface repelling water droplets, (**D**) contact angle of 172° for the superhydrophobic membrane disc surface, and (**E**) a planar sheet plate of porous titanium alloy, which shows a water droplet penetrating the superhydrophilic membrane surface.

Both planar and tubular forms of the porous inorganic membranes were evaluated in this study. Figure 3 shows inorganic membranes substrates made from a variety of materials, including ceramics such as alumina, and metals/alloys like SS434 and titanium. By design, the surface of the membrane can be tailored to be either superhydrophobic or superhydrophilic for various separation needs (i.e., membrane permeation preference either for polar (water) or for non/less-polar component [ethanol]). The pore size of the support substrate can be tailored by adjusting the sintering temperature or by coating an intermediate layer of the desirable pore size. For example, the inner wall of a 4-µm porous SS434 tube (Figure 3B) was coated with nanoporous (8-nm) gamma-alumina and then functionalized to be superhydrophobic by a chemical solution of perfluoro-terminated alkyl silane precursor (1H,1H,2H, 2H-perfluorodecyltrimethoxysilane, PDTMS). The SS434 support substrate surface was also directly deposited with Hydrophil-S aerogel silica nanoparticles to provide a textured superhydrophilic inner wall surface. For the planar membrane, diatomaceous earth powder was deposited on one side of the nanoporous (~60 nm avg.) alumina ceramic disc (Figure 3C) and then functionalized to be superhydrophobic (showing contact angle of 172°).

**Figure 4:**
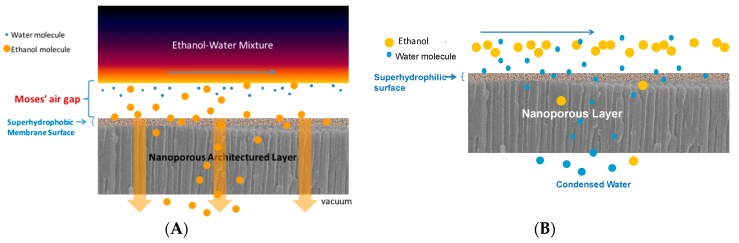
Schematic illustration of the separation principle of (**A**) superhydrophobic or (**B**) superhydrophilic membranes. The feed above the membrane surface can be either liquid or vapor mixtures.

Conceptually, hydrophobic-surfaced HiPAS membranes repel the polar liquid/molecules (such as water) while they attract the non-polar or less-polar liquid/molecules (such as ethanol) allowing them to permeate through the membrane pores (Figure 4A). Conversely, the hydrophilic surfaced membranes prefer polar liquid/molecules to penetrate into and pass through, while rejecting less-polar or non-polar liquid/molecules (Figure 4B). These two schematic figures will be used to explain the separation data presented later.

There have been research efforts on the development and advancement of superhydrophobic coatings [47,48,49]. However, they have not been developed for porous membrane applications. For most coating applications, it may not matter if the coating or underlying support substrate is permeable or not, but for membranes it does. Deposition of a superhydrophobic permeable coating layer on a permeable porous substrate to make a membrane represents a totally new challenge in material science. For example, it is much more difficult to make a thin permeable layer on the rough surface of a porous substrate than on a smooth surface of a nonporous solid substrate.

### 3.1. Vapor Phase Dehydration of Ethanol–water Mixtures with ~6 nm HiPAS Membranes 

**Figure 5:**
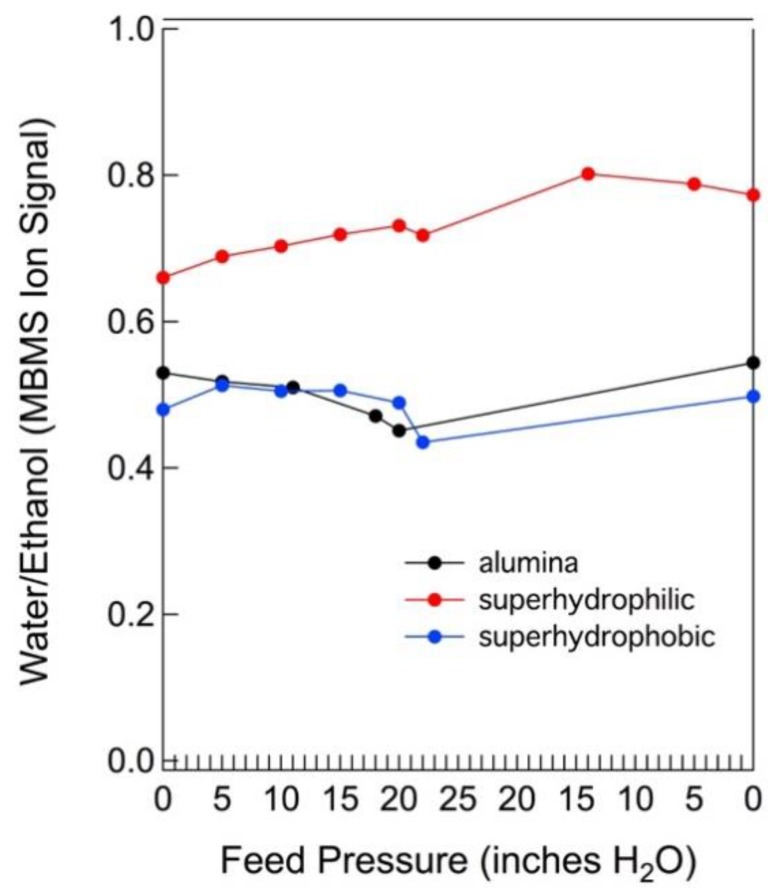
Evaluation of vapor phase ethanol–water separation selectivity for three types of membrane surfaces (alumina coated membrane, superhydrophilic, and superhydrophobic surface modified membranes [membrane #0, #8, and #12, respectively]). A molecular beam mass spectrometer (MBMS) was used to monitor the composition of the permeate as a function of feed pressure through a membrane.

Figure 5 shows the separation performance data for three different types of membranes using an ethanol–water vapor mixture. All three tested membranes have the same alumina-coated mesopores (~6 nm) on identical porous (4.3 µm) SS-434 support tubes but with different surface properties: bare alumina (no coating), superhydrophilic coating, and superhydrophobic coating. The tubular membranes were mounted in a holder (see Section 2) so that adjusting the metering valve on the raffinate stream controlled the feed pressure. The ethanol–water mixture (50/50 mixture by volume) was dispensed by a programmable syringe pump into an inert gas stream flowing through a horizontal quartz flow tube reactor heated to 200 °C. When vaporized, the gas stream contained approximately 1.88% water vapor by volume and 0.58% ethanol vapor. The concentrations of water and ethanol vapors were analyzed via an in-line molecular beam mass spectrometer (MBMS). The MBMS response was measured for the feed mixture and the ratio of the signal for water to that of ethanol was approximately 0.48. This vapor mixture was fed into the tubular membranes which were also held at 200 °C. The transport of water and ethanol vapors through the membrane (permeate side) was analyzed via the MBMS. Since the MBMS system operates under a slight vacuum, the permeate stream was always at less than atmospheric pressure. In order to vary the driving force for the membrane permeability, i.e., the pressure difference or transmembrane pressure across the membrane, the feed pressure was adjusted in the range from atmospheric pressure (represented as 0 inches of water in Figure 5) to approximately 22 inches of water above atmosphere and back down to zero. From these data, the alumina-coated tubular membrane and the superhydrophobic membrane performed in a similar manner (Figure 5, black circles and blue circles, respectively), except that at low pressure (near 0 inches feed pressure) the water/ethanol ratio for the hydrophilic-natured bare alumina-surfaced membrane is consistently higher than for the superhydrophobic membrane. This lower water/ethanol ratio for the superhydrophobic membrane may be due to the repellence of water by the superhydrophobic membrane surface. By comparing the water/ethanol ratio data between superhydrophilic and superhydrophobic membranes, the difference is larger throughout the entire range of transmembrane pressures. The significantly higher water/ethanol ratio for the superhydrophilic membranes may be attributed to the preferential/selective permeation of water to ethanol, due to surface superhydrophilicity.

Under the tested range of higher transmembrane pressure conditions (5–25 inches H_2_O), the superhydrophobic membrane surface appeared to have no significant effect on the perm-selectivity (i.e., not showing preferred permeation of ethanol while inhibiting water permeation). The ratio of the MBMS ion signals for water over ethanol on the permeate side, (R_I_)_Permeate_, varied from about 0.44 to 0.54 for both the bare alumina membrane and the superhydrophobic membrane. These R_I_ values for permeate are very close to the R_I_ values for the feed mixture (~0.48), indicating that both membranes showed little selectivity for ethanol or water vapor. However, it was observed that for both bare and superhydrophobic membranes the ratio (R_I_)_Permeate_ decreased as the feed pressure was increased, indicating that as the feed pressure was increased the ethanol flux increased slightly relative to the water flux. As the pressure increases, the interaction (adsorption) between the gas molecules and the surface is expected to increase. A visual inspection of a water droplet beading up on the inside surface of the superhydrophobic membrane after completion of these high temperatures tests shows the overall superhydrophobicity appears to be stable under the test conditions. However, the possibility of membrane defects (over-sized pores) or small areas of surface degradation into the bare alumina areas (defects) on the inner wall of the tube cannot be excluded. Either because of weak surface interaction under the test conditions or the presence of defects, the superhydrophobic surface effect on water/ethanol separations was not obvious in our experiment in comparison with the bare alumina-surfaced membrane. Such minor differences in membrane performance may be due to the observation that the hydrophilic-natured alumina-surfaced membrane contains hydrophobic spots (as confirmed by visual observation by placing a water droplet on the inner wall of the membrane tube). Additionally, the minor difference could be due to non-separative flow through over-sized pores. Separation resulting from Knudsen diffusion does not occur in large pores as the mean free path between molecules is decreased as the pressure increases.

For the superhydrophilic membrane, the values for the water/ethanol ratio (R_I_)_Permeate_ were in the range of 0.66 to 0.8 (Figure 5, red circles), which is higher than the bare alumina-coated membrane and superhydrophobic membrane. Using an average (R_I_)_Permeate_ of 0.76, the separation factor for water over ethanol was calculated using Equation (1), where *C* represents the concentration of the molecular species in the feed and permeate determined by MBMS, and found to be 1.53.

Separation Factor = (*C*_water permeate_/*C*_ethanol permeate_)/(*C*_water feed_/*C*_ethanol feed_) = (R_I_)_Permeate_/(R_I_)_Feed_ (1)

For a membrane, the mechanisms for gas (or vapor) selectivity can be due to many factors including Knudsen diffusion [50], size exclusion, surface diffusion, adsorption, and capillary condensation [51]. For membranes with no surface interaction, Knudsen diffusion and size exclusion are the two most commonly employed mechanisms for separation of gases. Size exclusion, or also sometimes referred to as molecular sieving, requires membrane pores that are on the order of molecular dimensions so that one molecule fits in the pores and transports through while the other molecule is too large to fit in the pores. Since the pores in these membranes are on the order of 6 nm and most gas molecules, including water and ethanol, are smaller than 0.5 nm, size exclusion is an unlikely separation mechanism for this separation. Knudsen diffusion employs the relative kinetic velocity of the gases for separation by a typical mesoporous membrane (2–50 nm diameter long narrow pores). When the gas density is low, the mean free path between molecules is much greater than the pore-size and Knudsen diffusion can be assumed as the gas molecules collide elastically (no surface interaction) with the pore walls more frequently than with each other. The theoretical perm-selectivity for Knudsen diffusion is the inverse ratio of the square root of the molecular weights.

*S*_water/ethanol_ = (MW_ethanol_/MW_water_)^1/2^ = 1.6 (2)

The separation factor from our experimental data, *S*_superhydrophilic_ = 1.53, is slightly less than the theoretical value (*S*_water/ethanol_ = 1.6) predicted by separation solely due to Knudsen diffusion. On the other hand, for the bare alumina membrane and superhydrophobic membrane, the separation factors, *S*_bare alumina_ and *S*_superhydrophobic_, were both around 1.0 and are much smaller than predicted by Knudsen separation alone, where *S*_water/ethanol_ = 1.6. Thus, Knudsen diffusion mechanism cannot explain the observed experimental data here. Note that capillary condensation phenomenon was not observed in the experiment. But, such a difference in water/ethanol ratio data (Figure 5) between superhydrophilic and superhydrophobic may be attributed to the adsorption and surface diffusion mechanism. In fact, HiPAS membranes are designed on the principle of membrane surface interaction with the liquid/molecules. Superhydrophilic surfaces should have a stronger attraction with polar molecules (water) than with less-polar molecules (ethanol); while superhydrophobic surface have a stronger repellence to water than ethanol. These strong surface interactions (attractive or repellant) appear to have affected the adsorption and surface diffusion processes.

While the selectivity is a property of the membrane and separation mechanism alone, the measured separation factor includes factors such as mixing, concentration polarization, and membrane cut (i.e., the percentage of product removal from the feed) and is almost always lower than the theoretical value of membrane selectivity defined by the mechanism (such as Equation (2)) under ideal conditions. Clearly, the superhydrophilic surface did affect the measured separation factor *S* relative to the non-modified bare alumina membrane. Therefore, the data indicates that at 200 °C, the superhydrophilic surface showed a Knudsen diffusion-like selectivity, while the non-modified, bare alumina-coated membrane shows no selectivity during transport of water-ethanol mixture. However, the surface adsorption/diffusion mechanism may not be excluded here. The interaction (absorption) of the molecules with the membrane surface increases as the temperature is decreased and lower temperatures may be needed in order to observe enhanced influence from the surface character. For the conditions tested here, the bare alumina-coated membrane and the superhydrophobic membrane showed little or no Knudsen selectivity. Little Knudsen diffusion would usually indicate viscous or non-separative flow, which is unlikely in the 6 nm pores. Thus, it is likely that surface adsorption/diffusion and/or oversized pores contributed to the lack of selectivity as would be predicted from Knudsen diffusion.

### 3.2. Liquid Separation by Pervaporation of Ethanol–Water Mixture

**Figure 6:**
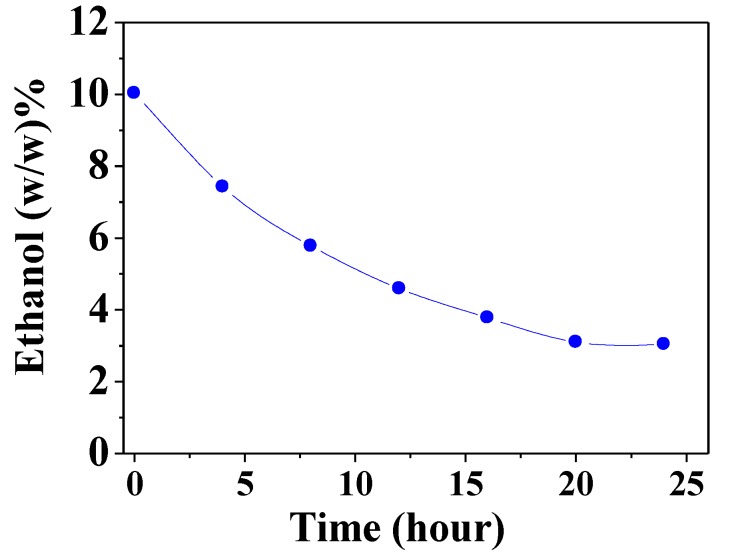
Liquid-phase separation of an ethanol–water solution with a superhydrophobic HiPAS membrane (SO disc). Ethanol concentration in the re-circulated feed stream as a function of time.

A nanoporous alumina ceramic-disc superhydrophobic membrane (172° contact angle, as shown in Figure 3C,D) having a pore size of ~60 nm was employed to separate an ethanol–water liquid mixture in pervaporation mode. A liquid feed solution containing 10 wt % ethanol was recirculated through the membrane side of the holder (Figure 2) and the solution was continuously diluted due to preferential ethanol permeation. The data in Figure 6 show that the ethanol concentration in the feed reservoir decreased significantly down to 2 wt % over a period of time while the feed continuously re-circulated in a flow-through configuration. As a baseline ceramic disc membrane (with no superhydrophobic surface functionalization), we observed no selective permeation of ethanol and thus show no feed ethanol concentration decrease with time as shown in Figure 6 for a superhydrophobic ceramic disc membrane.

Permeation through the membrane was achieved under pervaporation conditions at 80 °C. The collected condensate on the permeate side was ~30 wt% ethanol while the feed-side ethanol concentration was reduced down to 3% at the end of the experiment, indicating an approximate cumulative average perm-selectivity of 13.9 using the following equation:

Perm-selectivity = (*C*_Ethanol_/*C*_Water_)_Permeate_/(*C*_Ethanol_/*C*_Water_)_Feed_ = (30%/70%)/(3%/97%) = 13.9 (3)

where *C* is the concentration (wt %) in the solution. The values for feed were the average of the starting and ending concentration. The separation mechanism here is believed to be similar to the previous studies where pervaporation was used to remove organic molecules from water using conventional hydrophobic membranes [52,53,54,55]. Relative to conventional hydrophobic surfaces, the super hydrophobicity of HiPAS membranes may have a higher affinity for the organic (ethanol) molecules than for the water molecules. This affinity difference was visually observed during ethanol and water droplet penetration tests performed on the membrane disc surfaces having varying degrees of hydrophobicity and hydrophilicity. The ethanol droplet penetrated faster into the superhydrophobic disc surface than the less hydrophobic or hydrophilic disc surfaces. The data shown in Figure 6 demonstrate that a HiPAS membrane could be used to separate two molecules based on differences in polarity. In addition, the superhydrophobic HiPAS membrane employed in these tests had pores that are ~200× larger than those in a traditional zeolite membrane (~0.5 nm pore size). The high flux, ethanol-permeable HiPAS membrane could have a potential impact on the energy and processing efficiency of ethanol–water separations by extracting the smaller volume of ethanol from the water (rather than permeating the larger volume of water from the ethanol) typically found in a representative ethanol fermentation broth (e.g., 95% water and 5% ethanol). A conventional water perm-selective zeolite membrane must process the large volume of water to obtain >99.5% ethanol, while the ethanol perm-selective HiPAS membranes only needs to permeate the smaller quantity of ethanol in the mixture. Beyond our current proof-of-principle studies on superhydrophobic membranes to extract ethanol via pervaporation, future work will focus on improving the HiPAS membrane pore size/structure and increasing the degree of surface superhydrophobicity (e.g., a contact angle larger than 172 degrees) which may favor higher selectivity of ethanol permeation than water, with the ultimate goal of achieving higher flux performance for selective ethanol/water separation by increasing the pore size.

**Figure 7:**
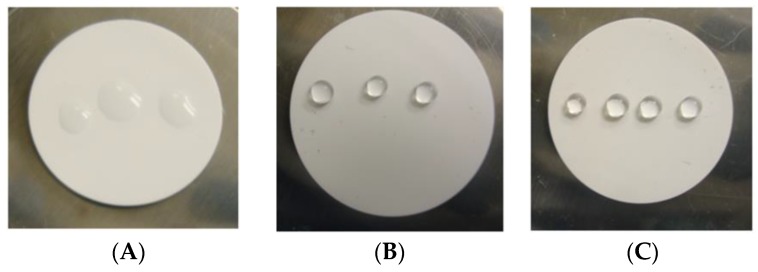

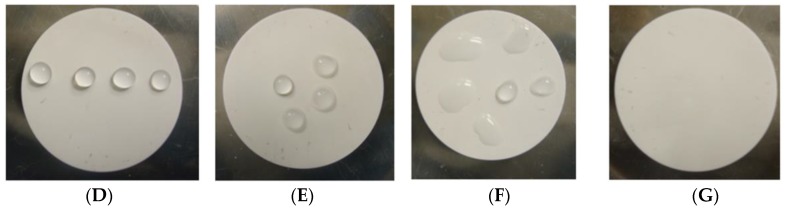
Surface hydrophobicity test to monitor the stability of wetting properties of a superhydrophobic membrane surface. (**A**) Blank alumina disc surface (hydrophilic in nature); (**B**) After PDTMS functionalization, showing superhydrophobicity (172° contact angle); (**C**) After PDTMS modification and heated at 300 °C for 2 h (still showing 172° contact angle); (**D**) After 300 °C for additional 4 h (172°); (**E**) After 350 °C for 2 h (136°); (**F**) 350 °C for additional 4 h (surface is wettable now with water droplets); and (**G**) 400 °C for 2 h (surface becomes so hydrophilic, and water droplets can penetrate into the disc surface and disappear).

Lastly, the chemical and thermal stability of HiPAS membranes (i.e., the coating and functionalization materials) were investigated. For superhydrophilic texture deposition, typical silica aerogel materials were inorganic materials, which do not display a stability concern for the application conditions of interest. For the superhydrophobic membrane materials surface, the observed water droplet contact angle was unaffected by the experimental conditions. Thus, exposure to ethanol–water or acid–water mixtures does not appear to cause observable degradation on the membrane surface hydrophobicity. In addition, thermal stability of superhydrophobicity of the membrane was studied by both visual inspection and contact angle measurements (Figure 7). Results show that the PDTMS-based superhydrophobicity of the membrane surface can be stable up to 300 °C, which is high enough for the example testing application conditions discussed in this paper.

## 4. Conclusions

This work reports on a novel surface-tailored HiPAS inorganic membrane concept where separations by superhydrophobic and superhydrophilic membranes were compared to baseline porous membranes without surface modification. (1) In the vapor-phase separation of ethanol–water, a superhydrophilic membrane demonstrated higher water/ethanol ratio in permeate samples than the superhydrophobic and baseline alumina-surfaced membranes. The potential separation mechanisms were discussed in relation to the collected experimental data. (2) In liquid-phase pervaporation separation studies on ethanol and water, a superhydrophobic membrane showed selective permeation of ethanol from the ethanol–water mixture feed. In both cases, results show that the surfaces of HiPAS membranes can be tailored for optimized separation of target molecule from a mixture.

## Figures and Tables

**Table 1:** Summary description of tested membranes.

Membrane Separation Performance Evaluation Tests		Membrane Types	Membrane Substrates	Surface Coatings	Membrane ID
Vapor Phase Separation	*Separations of ethanol–water vapors by membranes (~6 nm membrane pores)*	Bare alumina-coated support (hydrophilic)	4.3-µm porous SS434 tube	6-nm porous alumina + no surface ligand modification	#0 Baseline tube
		Superhydrophilic	4.3-µm porous SS434 tube	5.79-nm porous alumina + hydroxylated silica aerogel nanoparticle coating	#8 SI-tube
		Superhydrophobic	4.3-µm porous SS434 tube	6.4-nm porous alumina + diatomaceous earth (DE) coating +1H,1H,2H, 2H-perfluoro decyl trimethoxy silane (PDTMS) modification	#12 SO-tube
Liquid Phase Solution Pervaporation	*Pervaporative extraction of ethanol from ethanol–water mixtures (~60 nm membrane pores)*	Bare alumina disc (Hydrophilic surface)	Alumina ceramic disc with ~60-nm pores	DE coating + PDTMS modification → superhydrophobic surface	Baseline Disc
		Superhydrophobic	Alumina ceramic disc with ~60-nm pores	DE coating + PDTMS modification → superhydrophobic surface	SO Disc

## Abbreviations

HiPAS	High-performance architectured surface selective
SS	Stainless steel
SI	Superhydrophilic
SO	Superhydrophobic
PDTMS	1H,1H,2H, 2H-perfluoro decyl trimethoxy silane
MBMS	Molecular beam mass spectrometer
